# Role of laparohysteroscopy in women with normal pelvic imaging and failed ovulation stimulation with intrauterine insemination

**DOI:** 10.4103/0974-1208.63117

**Published:** 2010

**Authors:** K Jayakrishnan, Aby K Koshy, R Raju

**Affiliations:** Fertility Research and Gynecology Centre, KJK Hospital, Trivandrum - 695 015, Kerala, India

**Keywords:** Female infertility, intrauterine insemination, laparoscopy

## Abstract

**CONTEXT:**

Women with primary infertility and no obvious pelvic pathology on clinical evaluation and imaging are either treated empirically or further investigated by laparoscopy.

**AIMS:**

The role of diagnostic laparoscopy in women who fail to conceive after empirical treatment with ovulation induction and intrauterine insemination was evaluated.

**SETTINGS AND DESIGN:**

Retrospective study at a private infertility center.

**MATERIALS AND METHODS:**

A study of patients who underwent diagnostic laparoscopy between 1^st^ January 2001 and 31^st^ December 2008 was performed. Those patients who had no detectable pathology based on history, physical examination, and ultrasound and had treatment for three or more cycles in the form of ovulation induction and IUI were included in the study. Moderate and severe male factor infertility and history of any previous surgery were exclusion criteria.

**STATISTICAL ANALYSIS USED:**

Data were statistically analyzed using Statistics Package for Social Sciences (ver. 16.0; SPSS Inc., Chicago).

**RESULTS:**

Of the 127 women who underwent diagnostic laparoscopy and hysteroscopy, 87.4% (*n* = 111) of patients had positive findings. Significant pelvic pathology (moderate endometriosis, pelvic inflammatory disease, and tubal pathology) was seen in 26.8% of cases.

**CONCLUSION:**

One in four women had significant pelvic pathology where treatment could possibly improve future fertility. Diagnostic laparoscopy has a role in infertile women with no obvious abnormality before they proceed to more aggressive treatments.

## INTRODUCTION

Fertility investigations are often based more on tradition and personal preference than on the demonstrated usefulness of its components.[1] Among the many investigations available to evaluate the female partner of the infertile couple, laparoscopy is relatively recent. It has often been used in the evaluation of patients with infertility where other diagnostic methods have failed to come up with a cause. In addition, it has the advantage of being a ‘see and treat’ modality. Diagnostic laparoscopy, which is often combined with hysteroscopy, therefore, is frequently a standard procedure performed as the final test in the infertility work up in many clinics before the couple progresses to infertility treatment.[2]

Laparoscopy is considered to be the gold standard for the evaluation of the pelvis and is considered a safe procedure. It may improve pregnancy rates and quality of life. Costs of further fertility treatments may be reduced by enhancing response to treatment, guiding further management, circumventing treatments that are of low benefit and avoiding complications like multiple pregnancies.[3] This is though not without risks, and includes those inherent to the surgical procedure and the anesthesia administered.[4,5] The costs incurred and the risks involved in the procedure need to be balanced with the therapeutic benefit. Though laparoscopy has now become established as a preferred treatment modality for pelvic pathology, there have been areas of debate on its timing and use in the investigation of infertility. Improvement in imaging techniques has meant that both clinicians and patients would think twice before opting for an invasive procedure like diagnostic laparoscopy. Thus while laparoscopy used to be part of the basic infertility workup, it is now reserved for those cases where it is necessary to elaborate an identified pathology, or to define symptoms. It may then contribute significantly to subsequent management.[6]

There is a paucity of high-quality guidelines for treating infertile women with no obvious pelvic pathology and normal semen parameters; and management is often empirical.[7] These couples are usually either offered a short course of treatment or further evaluation in the form of laparoscopy. The former usually involves a few cycles of ovulation stimulation, which is often combined with intrauterine insemination (IUI).[8] Both clinicians and patients often face a dilemma if treatment fails. Further management options include a laparoscopy, if not already performed or progression to *in vitro* fertilization (IVF).

The increased availability, affordability, and success rates with IVF have questioned the need for laparoscopy in such women. Fatum *et al.*[8] have argued that in women with a normal HSG, the chances of detecting a peritoneal factor is very low and performing laparoscopy may be perceived as a waste of time and energy. They advise assisted reproductive technologies (ART) in such women rather than performing another expensive investigation. There is thus a growing tendency to overlook laparoscopy and proceed to ART in such patients. On the contrary, there is a view that women are increasingly being recommended to proceed to ART following an accelerated and often incomplete work-up.[9] It is important to understand that the costs of ART are high and each attempt usually offers only a single chance for pregnancy.

This study tries to evaluate the role of laparoscopy and hysteroscopy in women with an apparently normal pelvis who fail to conceive after they receive treatment in the form of ovulation stimulation with IUI. It tries to provide evidence to enable clinicians to counsel such couples with regard to the usefulness of laparohysteroscopy in their further management.

## MATERIALS AND METHODS

We reviewed case records of all patients who underwent laparoscopy and hysteroscopy for infertility between 1^st^ January 2001 and 31^st^ December 2008. All patients who met the following criteria were included in the study: (1) primary infertility defined as failure to conceive after 1 year of trying; (2) no detectable pelvic pathology based on history, physical examination, and transvaginal ultrasound; (3) ovulatory cycles confirmed on follicular tracking by transvaginal ultrasound-follicular rupture was confirmed by decrease in size and irregular shape of the leading follicle or by the characteristic appearance of the corpus luteum with diffuse internal echoes. Presence of fluid in the pouch of Douglas or hyperechoic endometrium was taken as supporting features; (4) Received treatment for three or more cycles in the form of ovulation stimulation with IUI. Exclusion criteria included history of any previous surgery and history of symptoms such as pelvic pain, severe dysmenorrhea, dyspareunia, and pelvic inflammatory disease. Couples with moderate and severe male factor infertility were also excluded.

Data were collected from patient case records in a data entry sheet. This included demographic factors such as age, duration and type of infertility, medical and surgical history symptoms as described in the exclusion criteria, clinical examination findings, gynecological ultrasound, and blood analysis for a baseline hormonal profile (day 2 serum follicle stimulating hormone, prolactin and thyroid stimulating hormone). Previous treatment history included details of ovulation stimulation, IUI and other treatment. Records of male evaluation were also noted and included medical history, clinical examination, sperm analysis (two separate samples with at least a 2-week interval and an interpretation according to World Health Organization (WHO) criteria).[10]

Intraoperative findings, surgical interventions, and operative complications during laparoscopy and hysteroscopy were noted. Presence of tubal obstruction, periadnexal adhesions, and endometriosis were recorded. In all cases, the severity of endometriosis was scored using the 1996 scoring system proposed by the American Society for Reproductive Medicine (formerly the American Fertility Society).[11]

Because this was a retrospective cohort study, informed consent by the patients was not needed. Specific approval by the institutional review board was taken before starting the study. Data were statistically analyzed using Statistics Package for Social Sciences (ver. 16.0; SPSS Inc., Chicago). Values are expressed as mean ± SD, unless otherwise stated.

## RESULTS

Although 2743 laparoscopic procedures were performed in infertile women during the 8 years of study period, only 127 (4.6%) of these patients fulfilled the eligibility criteria. These were the patients who had no positive signs on clinical evaluation or imaging and had received empirical treatment as stated above.

**Figure 1:**
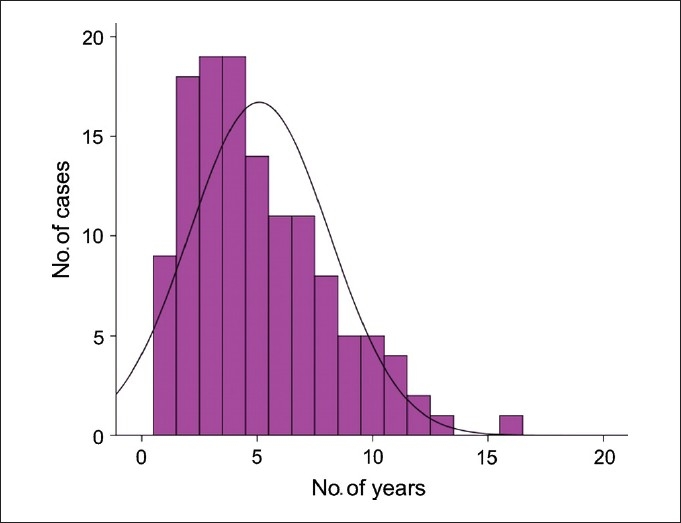
Mean duration of infertility

**Figure 2:**
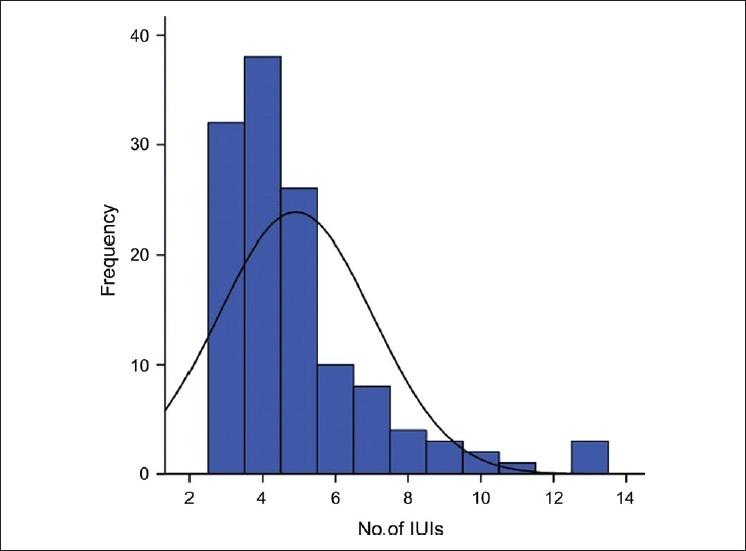
Number of cycles of ovulation stimulation with intrauterine insemination prior to laparoscopy

The mean age of the patients was 29.45 ± 4.34 years. The mean duration of infertility was 5.09 ± 3.03 years [Figure 1]. A mean of 4.91 ± 2.1 cycles of ovulation stimulation with IUI were performed prior to the surgical procedure [Figure 2]. Many women had more than three cycles of treatment prior to surgery, often at other fertility clinics.

The surgical findings on laparoscopy are displayed in Table 1. The major parameters assessed were the benefit of laparohysteroscopy in diagnosing significant pelvic pathology and assessment of tubal status. Of the 127 women, 12.6% (*n* = 16) had no detectable pathology on laparohysteroscopy. The incidence of endometriosis was 77.2% (*n* = 98); of which 48 (37.8%) patients had stage I, 42 (33.1%) had stage II, and 8 (6.3%) had stage III disease. No cases with Stage IV disease were present. There was one case of adenocarcinoma arising from endometriotic lesions. Thus, a majority of the patients had early stage disease (70.9%, *n* = 90).

**Table 1:** Surgical finding at laparoscopy

Finding at laparoscopy	Frequency	Percentage	Duration of infertility (years) (Mean ± SD)
All patients	127	100.0	5.09 ± 3.03
Normal	22	17.3	5.68 ± 2.57
Minimal endometriosis	48	37.8	5.04 ± 2.86
Mild endometriosis	42	33.1	4.86 ± 3.38
Moderate endometriosis	8	6.3	6.00 ± 3.74
Pelvic inflammatory disease	7	5.5	4.00 ± 2.64
Unilateral tubal block	17	13.4	6.06 ± 3.25
Bilateral tubal block	5	2.9	5.00 ± 4.06
Peri-tubal adhesions	7	5.5	5.14 ± 3.67

Note: Certain patients may have more than one positive finding

5.5% (*n* = 7) of patients had pelvic inflammatory disease (PID). This included one case of pelvic tuberculosis. 3.1% (*n* = 4) patients had unilateral and 2.4% (*n* = 3) had bilateral peritubal adhesions. On chromopertubation, 82.7% (*n* = 105) of patients had patent tubes, 13.4% (*n* = 17) had unilateral tubal block and 2.9% (*n* = 5) had bilateral tubal block. Treatment modalities at the time of laparoscopy included adhesiolysis, ablation of endometriotic deposits with bipolar diathermy, and hysteroscopic proximal tubal cannulation.

There were no abnormal findings at hysteroscopy. There were no major intra- or postoperative complications.

## DISCUSSION

Advances in imaging techniques have enabled accurate diagnosis of uterine and adnexal disease, thus redefining the role of laparoscopy. These techniques include two and three dimensional ultrasound, saline infusion sonography, computerized tomography (CT), and magnetic resonance imaging (MRI). Hysterosalpingography (HSG) and hysterosalpingo-contrast-sonography (HyCoSy) are inexpensive, fast, and well-tolerated methods of determining tubal patency, though their value when compared to laparoscopy is still a matter of debate.[12] These techniques have the advantage of being relatively noninvasive and less expensive compared to laparoscopy.

Diagnostic laparoscopy, which is often combined with hysteroscopy, is useful in ruling out Müllerian anomalies, revealing pelvic pathology, and assessing tubal function. In this era of improved imaging, the role of diagnostic laparoscopy, which is more invasive and expensive, has been questioned. This is especially so when initial clinical evaluation and imaging fail to find any abnormalities. The evidence for and against the use of laparoscopy in such cases has been inconsistent. In a retrospective study of 495 infertile women with unexplained infertility, laparoscopy before starting treatment revealed a significant incidence of abnormalities resulting in a changed treatment decision.[13] Among 172 patients (35%) with abnormal findings, 21 (4%) had severe abnormalities that resulted in a change of treatment to IVF or open surgery. In another 103 patients (21%), abnormalities like endometriosis (stages I and II), and adhesions were directly treated by laparoscopic intervention. The laparoscopic yield was lower if surgery done to remove early stage endometriosis is omitted - 40 out of 495 cases (8.1%). In a retrospective study of ovulation stimulation in 92 women, significant pelvic pathology was found in one-third of the patients failing to conceive after four ovulatory cycles of clomiphene citrate.[14] Most findings detected thus would not have been picked up by conventional imaging techniques. The authors concluded that early endoscopic diagnosis of such pathology would have allowed the couple better chances at future fertility and in selected cases to proceed directly to IVF. These findings though were not corroborated by other investigators. Lavy *et al*. assessed the diagnostic benefit of laparoscopy in 86 infertile women following hysterosalpingography (HSG). They concluded that laparoscopy may be omitted in women with normal HSG or suspected unilateral distal tubal pathology on HSG, since it was not shown to change the original treatment plan indicated by HSG in 95% of the patients. They however recommended laparoscopy in cases with suspected bilateral tubal occlusion on HSG, since it altered the original treatment plan in 30% of the patients.[15] al Badawi *et al*. retrospectively reviewed 265 women who had laparoscopies performed after normal hysterosalpingograms. Although 129 (49%) had one or more abnormal laparoscopic findings, only 7% of cases had findings that might require standard operative laparoscopy or laparotomy, although not all were causally related to infertility. They advocated a micro-laparoscopic approach for women where history and HSG were not suggestive of pelvic disease, reserving conventional laparoscopy for those with suspected pathology on HSG. They went on to suggest bypassing laparoscopy in favor of assisted reproduction in such selected cases as the perceived benefit of surgical intervention is small.[16]

Our study consisted of women with primary infertility who had a negative history, normal examination, and ultrasound findings and who failed to conceive with at least three cycles of ovulation stimulation combined with IUI. Although performing a hysterosalpingogram prior to IUI would have been ideal, it was not done in all patients and hence not included in the study. Only 4.6% of laparoscopies performed for infertility over an 8-year study period satisfied these criteria. Although the vast majority of patients had some positive findings at laparoscopy (87.4%, *n* = 111), it would be prudent to select those cases which would have findings significant enough to impair fertility and/or appreciably change further management. Capelo *et al*. defined 'positive laparoscopy' as surgical findings consisting of stages III or IV endometriosis, an endometrioma, pelvic adhesions, or tubal disease.[14] In this study, 26.8% of cases (*n* = 34) had a 'positive' laparoscopic finding. This included two uncommon diagnoses, viz. tuberculosis and adenocarcinoma arising from endometriosis.

There were no positive findings of hysteroscopy. This is not surprising as other authors have also demonstrated that a regular myometrial-endometrial interface and homogeneous endometrial structure on transvaginal sonography indicated a normal endometrium and precluded the need for diagnostic hysteroscopy.[17]

Performing a laparoscopy after at least three cycles of ovulation stimulation with IUI should have logically lead to a reduction in the number of negative laparoscopies, which is evident by comparatively higher detection of pelvic pathology in this study (87.4%). The number of women with significant pelvic disease as defined above was also fairly high compared to other studies (26.8%). These cases are those where laparoscopy would have lead to either increased fertility or a change in the treatment modality.

The benefit of laparoscopy in those women without ‘positive findings’, but with other minor pelvic pathology, is more contentious. Such cases accounted for 60.6% of the study population and largely included early stage endometriosis. A recent study showed that the likelihood of pregnancy was significantly reduced in infertile women with minimal or mild endometriosis compared with those infertile women with a normal pelvis.[18] Improved fecundity was seen with laparoscopic resection or ablation of minimal and mild endometriosis in a study of 341 infertile women,[19] though a smaller study involving 96 women did not show any benefit.[20] In addition to endometriosis, laparoscopy is also useful in releasing adhesions, especially peritubal ones which might impair ovum transport due to decreased tubal motility. Restoring normal anatomy might increase pregnancy rates, although the existing studies are nonrandomized.[21,22]

Demonstration of effectiveness of laparoscopy would be incomplete without it being cost effective. Unfortunately, there are insufficient studies to assess the cost-benefit ratio of laparoscopy in unexplained infertility. The Practice Committee of the American Society of Reproductive Medicine suggests that laparoscopy should be seriously considered before applying aggressive empirical treatments involving significant cost and/or potential risks.[23] In a cost-effectiveness analysis, a computer-generated decision analysis tree was used to compare expectant management, standard infertility treatment, and laparoscopy with and without infertility treatment. The study concluded that laparoscopy followed by expectant management is cost effective in the management of young couples with otherwise unexplained infertility.[3]

The timing of laparoscopy too has been a matter of debate. Although laparoscopy prior to initiating treatment looks attractive, the cost of this surgical procedure is high, especially when patients have to pay for the costs. Many clinicians thus prefer to treat couples with unexplained infertility with a few cycles of ovulation stimulation with IUI before proceeding to laparoscopy. A prospective randomized reallocation study to investigate the timing of laparoscopy after a normal hysterosalpingography was performed. This study, however, showed no significant difference in the prevalence of abnormalities with clinical consequences at laparoscopy before IUI when compared to laparoscopy after six cycles of IUI. The data suggested that the impact of the detection and the laparoscopic treatment of observed pelvic pathology prior to IUI seem negligible in terms of IUI outcome. The authors seriously questioned the value of routinely performing a diagnostic and/or therapeutic laparoscopy prior to IUI treatment.[24]

## CONCLUSIONS

Our study concludes that the use of laparoscopy in women with negative findings on clinical evaluation is of benefit, as at least a fourth of the women had conditions where treatment could improve future fertility. Another 60% of cases had findings where laparoscopic surgery might be of benefit. The role of routine hysteroscopy in these women requires further assessment.

Couples who fail to conceive with ovulation stimulation with IUI should be counselled that there is evidence to show that laparoscopy is of benefit before proceeding to ART. The use of empirical treatment in the form of ovulation stimulation and IUI prior to laparoscopy might reduce the number of patients requiring the procedure, reduce the number of negative laparoscopies, and optimize resource utilization. Further research in the form of prospective randomized multicenter studies and follow-up on pregnancy rates would be valuable.
